# Yoga for improving quality of life in contractual university teachers

**DOI:** 10.3389/fpubh.2024.1370426

**Published:** 2024-02-26

**Authors:** R. K. Roshni Raj Lakshmi

**Affiliations:** Department of Yoga, Manipur University, Imphal, Manipur, India

**Keywords:** contractual university teachers, job insecurity, quality of life, wellbeing, yoga

## Introduction

Capitalism has resulted in a brand of neoliberalism that espouses more flexibility, cost-effectiveness, and faster accomplishment (1). University teaching jobs worldwide are casualized to achieve the abovementioned goals. These contractual teachers are cheap, flexible, and disposable, as noted by Zheng (2). The university teaching faculties have a horizontal division and a vertical hierarchy. Due to the contractual nature of employment, these university teachers do not have access to certain benefits and facilities entitled to permanent university teachers. Certain unarticulated biases are introduced while dealing with the contractual employees from management, authorities, non-teaching staff, and teaching staff, often resulting in the marginalization of contractual university teachers (3, 4).

## The wellbeing of contractual university teachers

Job insecurity is one of the major causes of chronic stress among contractual university teachers (5). It is implicated as one of the major causes of adverse health conditions in contractual university teachers (6). Financial insecurity and bias in interacting with contractual or *ad hoc* teachers lead to chronic stress. Both psychological and physical dimensions of health are affected adversely by stress (7). Job insecurity and chronic stress in contractual university teachers are often associated with lifestyle disorders and mental health issues (5). Severe health issues affect the quality of life negatively (8). As explained by Maslow, several aspects of a human being's need are absent or insufficient for these contractual teachers, like employment, self-esteem, recognition, acceptance, and self-actualization. Maslow explained that each individual would feel anxious or tense if the needs were unmet, and he/she will not be motivated to strive for self-betterment (9).

## Yoga and wellbeing

Yoga is one of the six founding philosophies of ancient India known as shad darshanas. It has come to mean many things to many people. It is also a mind-body-medicine modality that has positively influenced health and its dimensions. It includes precepts, postures, breath regulations, meditation, diet regulations, and introspection. It is the “cessation of mental modifications” (1). Bhagavadgita defines yoga as “equanimity of the mind” (2). Several studies are available on individual practices and modules of yogic practices to improve many aspects of health (10). Yoga helps reduce stress by deactivating the Hypothalamus Pituitary Adrenal Axis (HPA) and Sympatho Adreno Medullary axis (SAM), reducing stress hormones, reducing interleukins and inflammatory agents, and activating the parasympathetic nervous system (11).

## Yoga as a panacea for improving the quality of life of contractual university teachers

Yoga is an effective mind-body medicine technique that improves the total health of an individual. For a contractual employee, many factors related to quality of life are influenced negatively. Material living is related to financial security, which is most significantly affected for contractual teachers. They are paid mainly by hours or with a lump sum with no increment or bonus for the same amount of labor. Social interactions differ drastically toward permanent and contractual teachers by management, colleagues, non-teaching staff, or students, often observed as a condescending or indifferent attitude (4). Fundamental rights are not similar for the two categories as some benefits like accommodation; other amenities are not accessible to a contractual teacher. Regarding the physical environment, the infrastructure allotted to a contractual teacher is congested and lacks ergonomic standards compared to the other category. Quality of life requires “a sense of belonging as an integral member of one's social network,” which is not the case for contractual university teachers as they are treated as the “others” in the academic institution (12).

Despite the disparities, yoga teaches the principle of contentment and the value of mental tranquility. The notion of *upeksha*, as explained by Patanjali as *chittaprasadanam*, explains that one should not get agitated and disturbed by bad events beyond his/her control as it will only destroy his/her mental tranquility (1). The Bhagavadgita espouses that a wise man is unaffected by both ups and downs of life (2). Patanjali yoga sutras explain that one should be satisfied with what he/she has at the moment (concept of *santosha*) (1), for peace and tranquility is above all wants and desires. Also, the practice of yoga techniques like postures, breath regulation techniques, and meditation are beneficial for improving mental resilience (13), self-esteem (14), emotional regulation (15), and clinical conditions (10) thereby improving overall health.

Yoga helps in inculcating positive values in an individual. It helps achieve spiritual health through introspection, awareness of the meaning of life, ethics, and building up the connection to the divine (16). Yoga also increases mindfulness (17), an essential prerequisite for mental wellbeing. Contractual university teachers are often stressed out due to a lack of time and excessive workload (3). Introducing mindfulness-based practices like meditation will improve resilience and cheerful temperament.

A yoga module can be designed including practices that reduce stress and enhance mental wellbeing and incorporated in the daily regimen of the university teachers. Yoga is found beneficial in management of stress and stress-related disorders (18). Practices like yogic postures, breath regulation, meditation, and relaxation techniques improve mental health by regulation HPA axis and stress response (19). Yoga is proven to reduce depression, anxiety and enhance quality of life in various groups of people (19, 20). Yoga is beneficial for both physical and mental health and both aspects are affected in these teachers (21). Quality of life is positively affected by yoga and hence yoga will be beneficial for this group of teachers (22). Thus, a yoga module incorporating practices that improve these aspects of mental domain which are affected in contractual university teachers should be developed and included in the health regimen of these teachers.

The article posits that yoga could be an effective tool for enhancing several aspects that influence the quality of life or, instead, change an individual's perspective to view the world with a new lens of positivity and strength (23). Mental resilience and spirituality, as learned in yoga, can thus improve the quality of life of contractual university teachers by reducing health issues stemming from chronic stress. Thus, yoga can be incorporated as an institutional practice of universities to improve the quality of life of university employees. Further research should be conducted to ascertain the probable benefits of yoga in the quality of life of contractual university (20) teachers using appropriate study designs. Longitudinal studies or stratified sampling studies should be conducted to observe the probable effect of yoga on the quality of life of contractual university teachers.

## Author contributions

RR: Conceptualization, Writing – original draft, Writing – review & editing.
